# Gene expression for secondary metabolite biosynthesis in hop (*Humulus lupulus* L.) leaf lupulin glands exposed to heat and low-water stress

**DOI:** 10.1038/s41598-021-84691-y

**Published:** 2021-03-04

**Authors:** Renée L. Eriksen, Lillian K. Padgitt-Cobb, M. Shaun Townsend, John A. Henning

**Affiliations:** 1 USDA Agricultural Research Service, Forage Seed and Cereal Research Unit, 3450 SW Campus Way, Corvallis, OR 97331 USA; 2 Department of Biochemistry and Biophysics, Oregon State University, Corvallis, OR 97331 USA; 3 Department of Crop and Soil Science, Oregon State University, Corvallis, OR 97331 USA

**Keywords:** Genetics, Agricultural genetics, Functional genomics, Gene expression, Plant breeding, Plant genetics, Plant sciences, Photosynthesis, Plant breeding, Plant genetics, Plant molecular biology, Plant physiology, Plant stress responses, Secondary metabolism

## Abstract

Hops are valued for their secondary metabolites, including bitter acids, flavonoids, oils, and polyphenols, that impart flavor in beer. Previous studies have shown that hop yield and bitter acid content decline with increased temperatures and low-water stress. We looked at physiological traits and differential gene expression in leaf, stem, and root tissue from hop (*Humulus lupulus*) cv. USDA Cascade in plants exposed to high temperature stress, low-water stress, and a compound treatment of both high temperature and low-water stress for six weeks. The stress conditions imposed in these experiments caused substantial changes to the transcriptome, with significant reductions in the expression of numerous genes involved in secondary metabolite biosynthesis. Of the genes involved in bitter acid production, the critical gene valerophenone synthase (VPS) experienced significant reductions in expression levels across stress treatments, suggesting stress-induced lability in this gene and/or its regulatory elements may be at least partially responsible for previously reported declines in bitter acid content. We also identified a number of transcripts with homology to genes shown to affect abiotic stress tolerance in other plants that may be useful as markers for breeding improved abiotic stress tolerance in hop. Lastly, we provide the first transcriptome from hop root tissue.

## Introduction

Secondary metabolites in hop (*Humulus lupulus* L.) cones provide the major flavoring agents in beer. Traditionally, the principle flavor was bitterness from alpha and beta acids, which are collectively known as the bitter acids. Other important flavor compounds include prenylated flavonoids such as xanthohumol, and volatile secondary metabolites or “hop oils”^1^. In addition, thiol precursors have recently attracted attention for their contribution to popular flavors^2–4^. Hop production in the United States was worth over $600 million in 2019. The state of Washington produced 73% of the country’s hops, and most of those hops were grown in the Yakima Valley^5^. Hop acreage in Washington is steadily increasing, however the yield per acre fluctuates yearly. In 2015, yield per acre declined^6^ during a period when Yakima county experienced abnormally dry to extreme drought conditions. Climate models for Washington state predict the coming decades will have decreased winter precipitation and an increase in the number of heat waves that will affect the Yakima Valley compared to previous decades^7^.

High temperatures and low-water stress during the growing season have consistently been shown to decrease hop cone yield and bitter acid content of cones^8–11^. A 25 year-long study in the Czech Republic found a positive correlation between yield and irrigation in cv. Saaz, Sladek, Premiant, and Agnus. They also found a significant negative correlation between summer air temperature and alpha acid content in cv. Saaz, Sladek, and Premiant, but not Agnus^10^. Mozny et al. also found decreased yield during low precipitation years and reduced alpha acid content in cv. Saaz hops during high temperature years in the Czech Republic^8^. Srečec et al. found similar reductions in yield and alpha acid content in cv. Aurora under low-water stress and heat stress in Croatia^9^. Nakawuka et al. in Washington state, U.S.A. found significantly decreased yield under reduced irrigation, but no significant effect on bitter acid content in cv. Mt Hood, Columbus, Chinook, and Willamette^11^. If climate model predictions prove true, hop production in Washington state could experience regular fluctuations in yield and in secondary metabolite content, which threaten the supply chain of a $116 billion beer market in the United States. There is thus increasing interest and need from hop growers to understand the response of hops to low-water and high temperature stress, and to develop new cultivars that have increased tolerance to abiotic stress.

The bitter acids and other flavor compounds are derived from secondary metabolites in the lupulin glands of the mature female inflorescences or “cones,” but these glands as well as the secondary metabolites therein are also present in leaf and stem tissues^12–14^. Bitter acids are prenylated polyketides that consist of alpha acids (humulone, cohumulone, and adhumulone) and beta acids (lupulone, colupulone, and adlupulone). These are derived from pyruvate precursors which are formed into the branched-chain amino acids (BCAA, i.e. leucine, isoleucine, and valine) via the BCAA biosynthesis pathway in the chloroplast^15^. The final step in the BCAA pathway is catalyzed by the enzyme branched-chain amino transferase 2 (*BCAT2*). For bitter acid biosynthesis, the BCAAs are then degraded in the mitochondria by branched-chain amino transferase 1 (*BCAT1*) and branched-chain keto-acid dehydrogenase (*BCKDH*) and converted by the enzyme valerophenone synthase (*VPS*) into phlorisovalerophenone (PIVP) in the cytosol^16–18^. An alternative pathway to PIVP synthesis is via the methyl-D-erythritol 4-phosphate (MEP) pathway in the chloroplast^19^. The PIVP is then prenylated by two prenyltransferases (*HlPT-1/HlPT1L* and *HlPT-2*^20–22^) in the chloroplast to the bitter acid precursor. The precise precursor molecule depends on the BCAA precursor, i.e. leucine, isoleucine, or valine. The final step of alpha acid synthesis remains unconfirmed to our knowledge, but is considered to involve conversion of that precursor to one of the alpha acids by deoxyhumulone hydroxylase, or humulone synthase. The final step of beta acid synthesis involves a third prenylation by *HlPT-2*^22^).

For xanthohumol biosynthesis, phenylalanine is converted through the p-coumaroyl-CoA and flavonoid biosynthesis pathways to p-coumaroyl-CoA, which is then adjusted by the enzyme chalcone synthase (*CHS_H1*^18^) and a chalcone isomerase-like protein (*CHIL2*^23^) to chalconaringenin. A prenyltransferase (*HlPT1L*^23^) converts chalconaringenin to desmethylxanthohumol, which is then methylated by an o-methyltransferase (*OMT1*^24^) to xanthohumol. Conversion of the PIVP precursor to PIVP by *VPS* is a critical step in the production of bitter acids, and total expression of *VPS*^25^ and *CHS*^16^ during cone development appears to correlate with bitter acid content among cultivars.

Volatile secondary metabolites such as terpenoids, isoprenoids, or “hop oils” also provide important flavors. The primary volatiles are monoterpene or sesquiterpene compounds that may be hydrocarbons, or they may be oxygenated or sulphinated^26^. These volatiles include the monoterpene myrcene, the sesquiterpenes alpha humulene and beta-caryophyllene, monoterpene alcohols such as linalool and geraniol^27^, and approximately 200 other compounds^1,28^. These compounds are produced from precursors derived from the MEP pathway, and then converted by specific prenyltransferases to geranyl diphosphate (GPP). Geranyl diphosphate is in turn converted to beta-myrcene by a monoterpene synthase (*MTS2*^29^), or farnesyl diphosphate (FPP) by squalene/phytoene synthase or farnesyl-PP synthase^30^, and thence to caryophyllene and humulene by sesquiterpene synthase 1 (*HlSTS1*^29^). Oxygenated volatile secondary metabolites include the monoterpene alcohols such as linalool, which are formed from GPP by S-linalool synthase^31^.

Volatile thiols are considered responsible for popular “tropical,” or “passion fruit” flavors in beer. These compounds are derived during the fermentation process by the action of yeast beta-lyase on non-volatile cysteine- or glutathione-S-conjugate precursors^32–34^. The precursors of thiol compounds have been identified in hop cones, and include glutathionylated and cysteinylated 4-methyl-4-mercaptopental-2-one (4MMP or 4MSP), and glutathionylated 3-mercaptohexan-1-ol (3MH or 3SH)^2,35^. The biosynthesis of these precursor compounds in plants is not well understood^36^, but may result from the conjugation of glutathione and 2-hexenal via glutathione S-transferase (*GST*)^37,38^, followed by conversion to S-3-(hexan-1-ol)-cysteine by membrane-associated gamma-glutamyl transferase carboxypeptidases (*GGT*)^38^. Additional flavor compounds include polyphenols such as carboxylic acids and non-prenylated flavonoids such as proanthocyanidins and flavonol glycosides^26^.

We measured physiological traits and used RNA-seq to understand the response of cv. USDA Cascade to high temperature (HT) stress, low-water (LW) stress, and a compound treatment of HT and LW stress, in comparison to control temperature (CT) and control water (CW) treatments. We used a split-plot experimental design exposing plants to control temperature and control water (CT/CW or control treatment), high temperature and control water (HT/CW or HT treatment), control temperature and low water (CT/LW or LW treatment), and finally a compound stress treatment of high temperature and low water (HT/LW). The project contributes to the genomic understanding of hop, and represents the first published transcriptome from root tissue in hop. The goal of this study was to understand the baseline response of *H. lupulus* to HT stress, LW stress, and a combination of the two stress factors*,* with particular attention to the genes involved in agronomically important secondary metabolite biosynthesis discussed above. The purpose was to identify general patterns and candidate genes for screening additional cultivars and breeding lines for increased abiotic stress tolerance.

## Results

### Trait phenotypes

**Figure 1:**
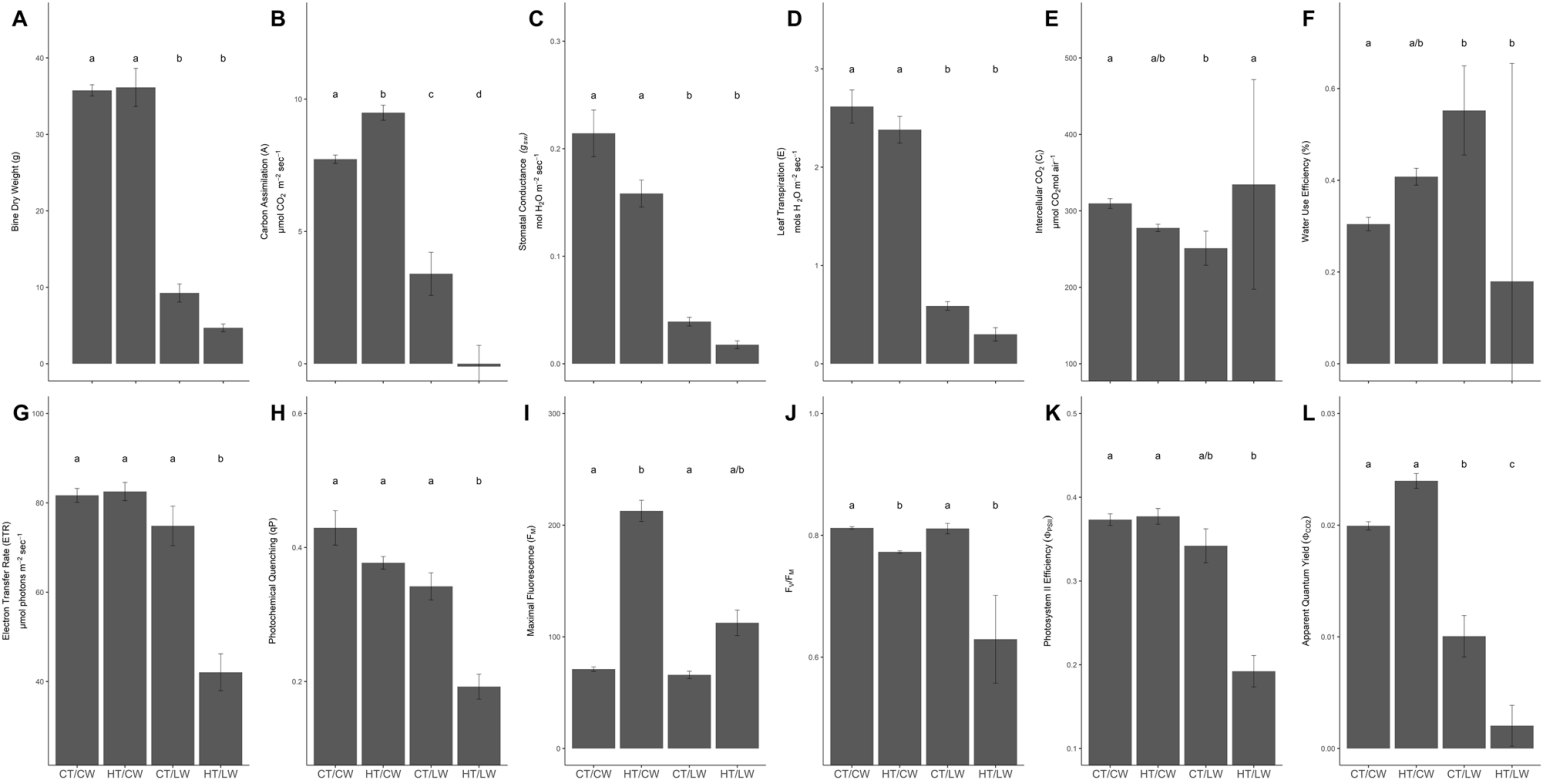
The average values of measured morphological and physiological traits taken prior to RNA-seq sampling. Letters above the bars represent statistical significance from ANOVA and Tukey HSD tests or Kruskal–Wallis and Dunn-tests. Error bars represent the standard error of the mean. (**A**) Bine dry weight (DW); (**B**) carbon assimilation (**A**); (**C**) stomatal conductance (*g*_*sw*_); (**D**) leaf transpiration (**E**); (**E**) Intercellular CO_2_ (C_*i*_); (**F**) water use efficiency (WUE); (**G**) electron transfer rate (ETR); (**H**) photochemical quenching (qP); (**I**) maximal fluorescence (F_M_); (**J**) F_V_/F_M_; (**K**) photosystem II efficiency (ɸ_PSII_); (**L**) apparent quantum yield (ɸ_CO2_).

Low-water (LW) stress affected bine dry weight (DW) more than temperature stress; DW was significantly lower in LW treatments than control and HT treatments (Kruskal–Wallis χ^2^ = 10.8, df = 3, P = 01) (Fig. 1A). Carbon assimilation (A) was significantly different among treatments (F_(3,13)_ = 91.4, P < 0.001), and highest under HT/CW (Fig. 1B). Stomatal conductance (*g*_*sw*_) (F_(3,13)_ = 21.8, P < 0.001) (Fig. 1C) and transpiration (E ) (F_(3,13)_ = 44.9, P < 0.001) (Fig. 1D) was significantly reduced in LW treatments (i.e. CT/LW and HT/LW). Intercellular carbon concentration (C_*i*_) was only significantly different among the CT/CW and CT/LW (Dunn test P = 0.006) (Fig. 1E). Water use efficiency (WUE) was significantly lower in CT/CW (F_(3,13)_ = 12.2, P < 0.001) but varied considerably in HT/LW treatments (Fig. 1F).

The electron transfer rate (ETR) (Kruskal–Wallis χ^2^ = 13.0, df = 3, P = 0.07) (Fig. 1G) and photochemical quenching rates (qP) (F_(3,13)_ = 14.7, P < 0.001) (Fig. 1H) were lower only in the combined stress HT/LW, but not among other treatments (ETR: Dunn test P > 0.10, qP: Tukey HSD P > 0.07). Maximal fluorescence (F_M_) was significantly increased under HT treatments (Kruskal–Wallis χ^2^ = 13.5, df = 3, P = 0.004) (Fig. 1I). F_V_F_M_ ratios, as a measure of maximum efficiency of photosystem II (PSII)^39^, were significantly reduced under HT treatments (Kruskal–Wallis χ^2^ = 7.5, df = 3, P = 0.004) (Fig. 1J), particularly under combined HT/LW treatments. Photosystem II efficiency (ɸPS2) was only significantly reduced under the compound stress HT/LW (Dunn test P = 0.01 for comparisons with HT/LW and both control water treatments) (Fig. 1K). Apparent quantum yield (ɸCO_2_) was significantly lower in LW treatments (F_(3,13)_ = 90.6, P < 0.001). The compound stress HT/LW reduced ɸCO_2_ to very low levels (Fig. 1L).

### Transcriptome

Libraries from leaf tissue produced an average of 23.8 million reads with 43.8% GC content. Approximately 77.9% of the libraries were duplicate sequences. The alignment of the raw reads to the masked, deduplicated primary genome assembly of ‘Cascade’^40^ achieved an average mapping rate of 62% ± 0.03 of the reads among libraries. Libraries from root tissue exposed to low-water stress produced an average of 49.8 million reads.

### Differentially expressed genes (DEGs) in leaf tissue: transcripts common among all treatments

**Figure 2:**
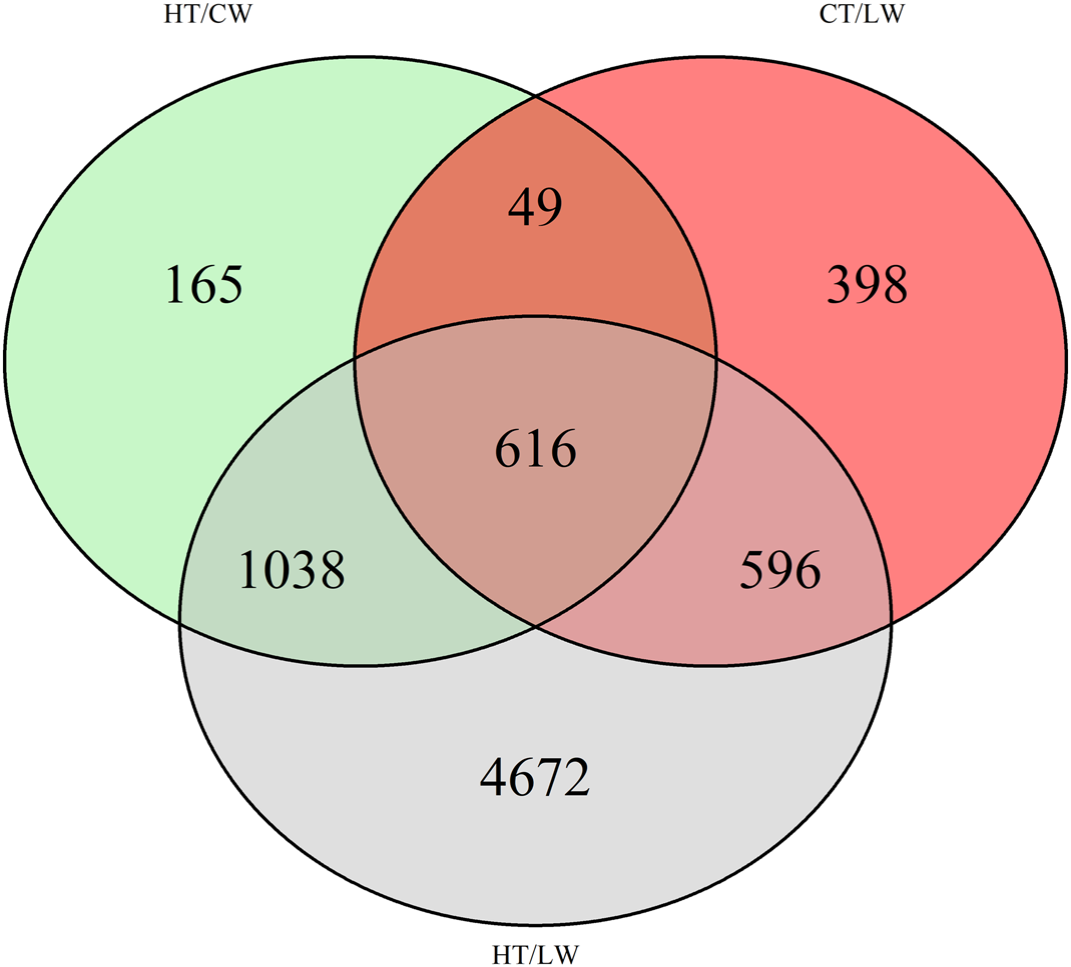
Venn diagram of the numbers of differentially expressed transcripts compared to control treatments (CT/CW).

A total of 616 transcripts were differentially expressed (DE) in all treatments compared to controls (Fig. 2); 169 of these were consistently down-regulated under stress treatment, and 447 were consistently up-regulated under stress treatment. Among the transcripts most down-regulated under stress treatments are a number of MADS-box transcription factors, two putative VPS transcripts, and a putative chalcone synthase transcript. Among those most up-regulated under stress treatment is a putative chemokine ligand 4, a putative disease resistance protein, and two putative ionotropic glutamate receptor transcripts.

There were 226 GO terms significantly enriched among the list of 616 differentially expressed genes (DEGs) common to all treatments compared to controls. Among the significant terms were several oxidation–reduction related categories (GO:0055114, GO:0016491, GO:0016701, GO:0016705, GO:1990204, GO:0098869, GO:0042743, GO:0042744, GO:0016209, among others), and several DNA replication related categories (GO:0006261, GO:0006270, GO:0006260, GO:0003896). Several terms associated with genes involved directly and tangentially in the biosynthesis of bitter acid and flavor production compounds were also enriched, including carboxylic acid biosynthesis processes (GO:0046394, GO:0072330, GO:0019752), BCAA metabolic processes (GO:0009081, GO:0052654, GO:0052655, GO:0052656, GO:0004084), and terpene synthase activity (GO:0010333).

### DEG in leaf tissue: transcripts in bitter acid biosynthesis pathway

We identified 43 transcripts that are homologous to known plant genes that code for proteins in the bitter acid biosynthesis pathway. This set included transcripts putatively involved in the BCAA biosynthesis pathway, the BCAA degradation pathway, VPS, the MEP pathway, and humulone synthase (Table 1). Four expressed transcripts are putative homologs to subunits of branched-chain aminotransferase (BCAT2 or 1); these four transcripts appear more likely to be BCAT2 which is involved in BCAA biosynthesis, than BCAT1 which is involved in BCAA degradation in the mitochondria, based on the limited homology to A*rabidopsis thaliana* BCAT1.

**Table 1:** A list of the pathways and genes involved in bitter acid (alpha and beta acids) production.

Pathway	Function	*Arabidopsis* gene count	*Humulus lupulus* transcript count	*Humulus lupulus* transcript
BCAA synthesis	Acetolactate synthase (ALS), catalytic subunit	1	2	000695F.g6, 002979F.g3
	Acetolactate synthase (ALS), regulatory subunit	2	4	001204F.g18, 001843F.g17, 003044F.g33, 003044F.g36
	Ketol-acid reductoisomerase (KARI)	1	2	005419F.g2, 005419F.g9
	Dihydroxy-acid dehydratase (DHAD)	1	1	000203F.g611
	Isopropylmalate isomerase (IPMI) heterodimer, large subunit	1	1	001924F.g4
	3-Isopropylmalate dehydrogenase (3-IPMD)	3	3	000238F.g111, 000854F.g10, 003837F.g22
	Branched-chain aminotransferase (BCAT2 or 1)	6	4	001480F.g11, 002627F.g2, 004331F.g5, 006224F.g8
BCAA degradation	Branched-chain alpha-keto acid dehydrogenase (BCKDH) Complex, E1 2-oxoisovalerate dehydrogenase subcomplex, subunit alpha	2	3	002778F.g14, 003232F.g12, 005302F.g1
	Branched-chain alpha-keto acid dehydrogenase (BCKDH) Complex.E1 2-oxoisovalerate dehydrogenase subcomplex.subunit beta	2	2	002884F.g31, 005146F.g17
	Branched-chain alpha-keto acid dehydrogenase (BCKDH) Complex.dihydrolipoamide dehydrogenase component E3	2	4	000063F.g43, 003977F.g21, 004663F.g10, 004663F.g14
MEP pathway	1-Deoxy-D-xylulose 5-phosphate synthase (DXS)	3	4	000003F.g120, 000442F.g55, 002303F.g16, 008968F.g1
	1-Deoxy-D-xylulose 5-phosphate reductase (DXR)	1	2	000825F.g9, 007147F.g3
	4-Diphosphocytidyl-2-C-methyl-D-erythritol synthase (CMS)	1	1	002759F.g2
	4-Diphosphocytidyl-2-C-methyl-D-erythritol kinase (CMK)	1	1	005048F.g3
	2-C-methyl-D-erythritol 2,4-cyclodiphosphate synthase (MCS)	1	2	001734F.g48, 003317F.g29
	4-hydroxy-3-methylbut-2-enyl diphosphate synthase (HDS)	1	1	002966F.g9
	4-hydroxy-3-methylbut-2-enyl diphosphate reductase (HDR)	1	1	001962F.g19
Bitter acid synthesis	Valerophenone synthase (VPS)		2	001329F.g74, 002397F.g29
	Humulone synthase		3	010673F.g1, 008118F.g14, 010625F.g1

The number of Arabidopsis thaliana genes is given according to Mercator4 annotations, and the number and transcript identifiers from *H. lupulus* cv. Cascade that are also according to Mercator4. Transcripts with low read counts (< 10) in leaf tissue were removed.

**Figure 3:**
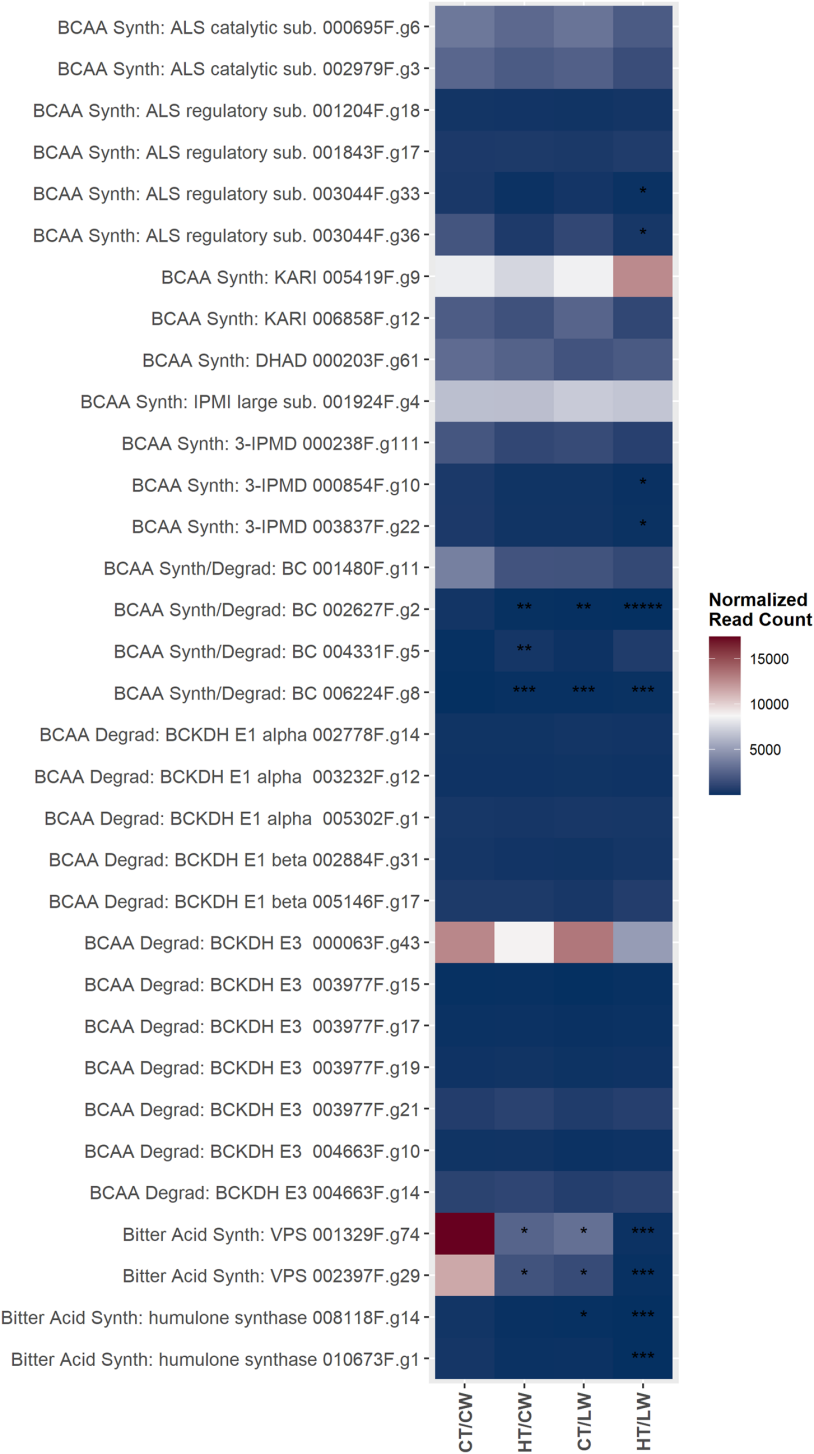
A heatmap of normalized read counts for transcripts putatively within the pathways to bitter acid production. Asterisk (*) indicate the log-fold change: * > 2 log2-fold change, ** > 4 log2-fold change, *** > 6 log2-fold change. Normalized read counts are not scaled across treatments per transcript. *ALS catalytic/regulatory sub* acetolactate synthase catalytic or regulatory subunit, *BCAA Synth* branched-chain amino acid synthesis, *BCAA Degrad* branched-chain amino acid degradation, *BC* branched-chain aminotransferase, *BCKDH* branched-chain alpha-keto dehydrogenase complex, *Bitter Acid Synth* bitter acid synthesis, *CMS* 4-diphosphocytidyl-2-C-methyl-D-erythritol synthase, *DHAD* dihydroxy-acid dehydratase, *DXS* 1-deoxy-d-xylulose 5-phosphate synthase, *DXR* 1-deoxy-D-xylulose 5-phosphate reductase, *IPMI large sub.* isopropylmalate isomerase (IPMI) heterodimer, large subunit, *3-IPMD* 3-isopropylmalate dehydrogenase (3-IPMD),* IVD* isovaleryl-CoA-dehydrogenase, *KARI* ketol-acid reductoisomerase, *VPS* valerophenone synthase.

Expression levels of transcripts involved in the bitter acid synthesis pathway in leaf and stem tissues were relatively low, and most did not change significantly among treatments (Fig. 3). There were substantial reductions in expression levels through all stress treatments in both putative VPS transcripts. Expression of one putative VPS transcript (001329F.g74) declined from an average normalized read count of greater than 17,000 reads in CT/CW treatments to 121 reads in leaf tissue exposed to the compound stress. There were also significant declines in expression levels in a putative BCAT2 transcript (002627F.g.2) across treatments.

The final steps of bitter acid production involve the conversion of PIVP in two or three prenylation reactions by prenyltransferases. Significant orthologs of published *HlPT1L* and *HlPT2* sequences were not recovered in transcripts from leaf, stem, or root tissue. For alpha acid production, precursors are reduced by humulone synthase. Expression of two transcripts orthologous to published humulone synthase declined significantly with >2 log2-fold change under LW stress and compound stress.

### DEG in leaf tissue: transcripts in volatile “oils” and thiol biosynthesis pathways

**Figure 4:**
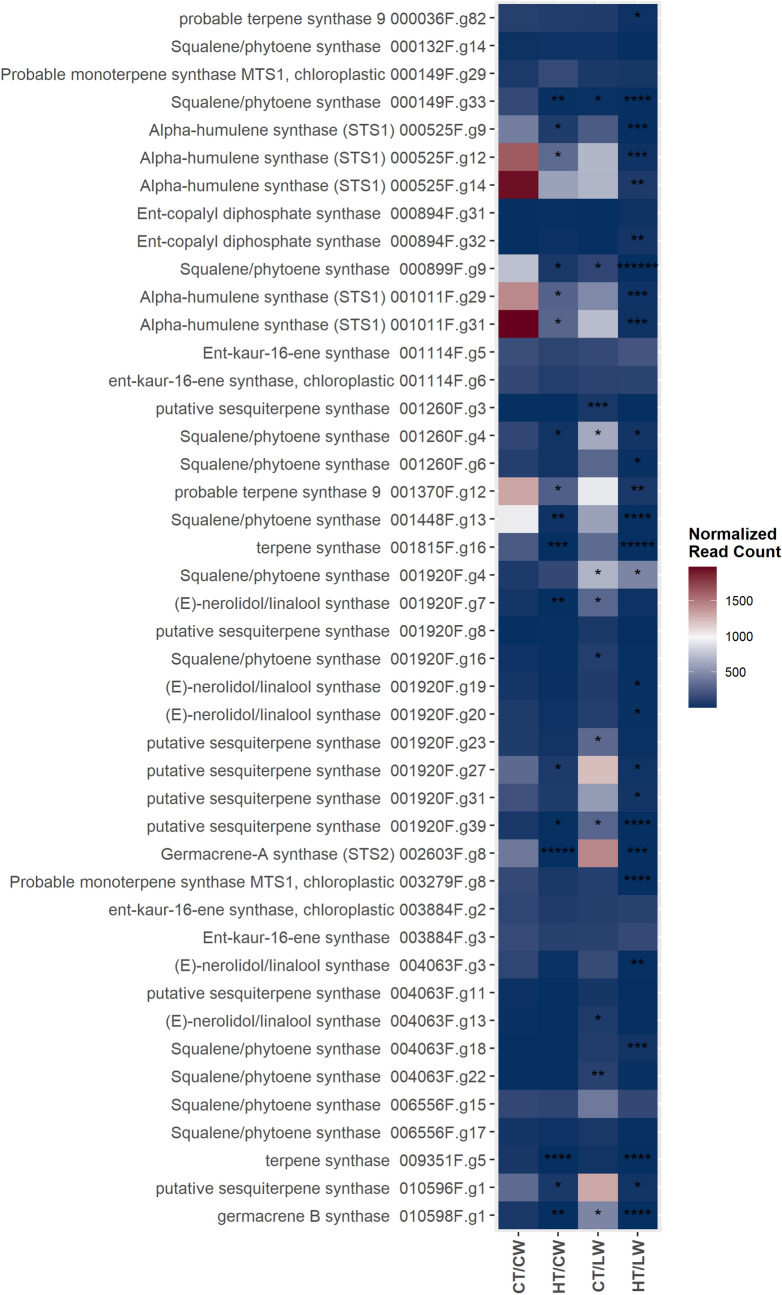
A heatmap of normalized read counts for transcripts putatively within the GO:0010333, terpene synthase activity. Asterisk (*) indicate the log2-fold change: * > 2 log2-fold change, ** > 4 log2-fold change, *** > 6 log2-fold change.

We identified 44 transcripts as putatively involved in biosynthesis of volatile secondary metabolites, or “hop oils,” including two monoterpene (myrcene) synthases, five putative humulene synthases, 12 squalene/phytoene synthases or farnesyl-diphosphate farnesyltransferases, and five nerolidol/linalool synthases (Fig. 4). Most of the humulene synthase transcripts were significantly down-regulated under HT and/or LW, as were many of the putative squalene/phytoene synthase and sesquiterpene synthases. There were also a large number of transcripts that were up-regulated under LW stress, including many putative sesquiterpene synthases, putative squalene/phytoene synthases, and putative nerolidol/linalool synthases (Fig. 4).

**Figure 5:**
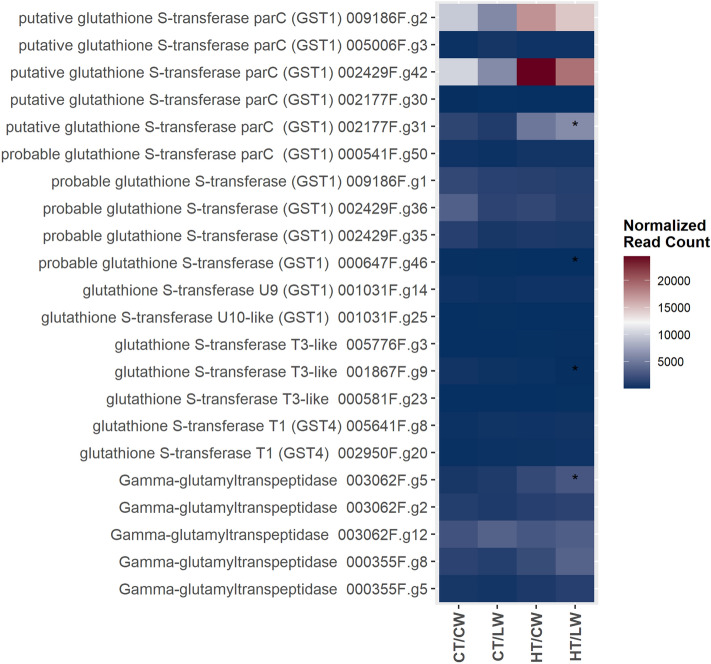
A heatmap of normalized read counts for transcripts putatively identified as glutathione S-transferase, which may contribute to varietal thiol precursor biosynthesis in hop cone. Asterisk (*) indicate the log2-fold change: * > 2 log2-fold change, ** > 4 log2-fold change, *** > 6 log2-fold change.

We identified 17 transcripts as putative glutathione S-transferases that may be involved in 3MH and/or 4MMP production. All but two of these transcripts are putative homologs of GST1, which is not associated with 3MH biosynthesis in grapes^37^. We also identified five transcripts with strong homology to GGT. For most of these transcripts, there were no significant differences in expression patterns in leaves among treatments. For three putative GST1 transcripts (002177F.g31, 001867F.g9, 000647F.g46) and one putative GGT transcript (003062F.g5), there was a significant increase in expression under HT/LW stress (Fig. 5).

### DEG in leaf tissue: controls × high temperature stress (CT/CW × HT/CW)

There were 1869 DEGs in comparisons of HT and control samples. Of the transcripts most significantly up-regulated is a putative chemokine ligand 4 (CCL4), a fatty acyl-CoA reductase, as well as several putative dehydrins and heat shock proteins. A number of putative serine/threonine protein kinases, a germacrene-A synthase, several terpene synthases, and four chalcone synthases transcripts were significantly down-regulated. Expression of the two putative VPS transcripts declined by approximately two-fold compared to controls, however these declines were marginally outside of statistical significance among treatments (P-adj = 0.08).

There were 974 GO terms enriched in comparisons of CT/CW × HT/CW, most of which were in the biological process category. Among them were more terms related to oxidation–reduction (GO:0055114, GO:0016491, GO:0016701, GO:1990204, GO:0016209, among others), as well as the terms for photosystem II (GO:0009523, GO:0009654).

One of the primary sites of damage induced by HT stress in a plant is the oxygen-evolving complex (OEC) of photosystem II^41,42^. A number of transcripts putatively coding for PSII D1 proteins were up-regulated under HT stress compared to controls, as were other transcripts related to PSII, including a putative PSII lipoprotein Psb28. Among these transcripts, many are up-regulated to a greater extent under the compound stress than under HT stress alone, though the putative D1 proteins were down-regulated under the compound stress. There were also a number of transcripts coding for putative antioxidant proteins involved in stabilizing PSII such as zeaxanthin epoxidase, vitamin K epoxide reductase, lipocalins that were up-regulated under heat.

Another primary site of heat damage is the process of carbon assimilation^41,43^. Our transcript database contained three transcripts each that match to the Rubisco large and small subunit, and three transcripts that match to Rubisco ATP-dependent activase. One transcript of the small subunit was significantly down-regulated under HT, LW, and compound stress compared to the controls. Two transcripts of putative Rubisco activases were up-regulated under HT compared to the controls.

### DEG in leaf tissue: controls × low-water stress (CT/CW × CT/LW)

There were 1661 DEGs in comparisons of LW stress and control samples. Of these, 598 were down-regulated, and 1601 were up-regulated. The most down-regulated transcripts include a putative 40S ribosomal protein, a ferredoxin, and several transcription factors. Both transcripts identified as VPS were significantly down-regulated, as well as two putative chalcone synthase transcripts, and a putative humulone synthase 2. Among the transcripts that were most up-regulated under LW stress include the putative butenolide signaling repressor, a peroxidase, and a carbonic anhydrase. A putative sesquiterpene synthase was up-regulated by > 7log2- fold. Several putative dehydrins and late embryogenesis abundant (LEA) proteins are also up-regulated.

There were 1041 GO terms that were significantly enriched in comparisons of CT/CW × CT/LW, including oxidation–reduction related processes (GO:0055114, GO:0098869, GO:0006979, GO:0034599, GO:0072593, GO:0006801, GO:0000305, GO:0019430 GO:0000303, GO:1990204, GO:0016491, GO:0016209, GO:0016667, among others). Enriched terms also included the MAPK signaling pathway (GO:0000165), and response to water deprivation (GO:0009414). Ten transcripts that are putative matches for dehydrins and LEA proteins were up-regulated under LW stress. Categories related to secondary metabolite production were also enriched, including BCAA biosynthetic processes (GO:0009082, GO:0009081), and carboxylic acid metabolic processes (GO:0019752).

Low-water stress response is mediated through ABA-dependent and ABA-independent pathways^44,45^. There are six members of the PYR/PYL/RCAR protein family that bind ABA and a protein phosphatase 2C. *Arabidopsis* has 29 genes that code for the subunits of these proteins, while there are 33 transcripts in our database from *H. lupulus*. There are 10 putative SnRK2 transcripts in our transcript database, but only five that are significantly differentially expressed with higher expression under LW stress. We identified 40 transcripts with significant homology to the *Arabidopsis* group-A bZIP transcription factors; six of these transcripts were significantly differentially expressed among CT/CW × CT/LW treatments, and also up-regulated under LW stress.

ABA-independent signaling is mediated through members of the AP2/ERF family DREB2A and DREB2B. We identified 142 transcripts that were significant matches to published DREB2A and DREB2B, two were significantly up-regulated under LW stress. *Arabidopsis* has one copy of GRF7, a DREB2 suppressor, however we identified six transcripts that are annotated as GRFs that are up-regulated under control conditions compared to LW treatments.

### DEG in leaf tissue: controls × compound stress (CT/CW × HT/LW)

The combination of the two stress factors, HT stress and LW stress, had a greater effect on gene expression than the cumulative effect of each stress factor. The greatest number of DEGs was in the comparison between CT/CW and combined stress HT/LW (Fig. 2). Among the most significantly down-regulated genes there were two putative 40S ribosomal protein transcripts, a BCAT2, a prenyltransferase, the two VPS, four chalcone synthase, nine monoterpene or sesquiterpene synthases, and five alpha-humulene synthase transcripts. The most up-regulated transcripts include a putative cytochrome P450 monooxygenase, a wall-associated receptor kinase, a carbonic anhydrase, six dehydrins, and two LEA protein transcripts.

There were 979 GO terms with significant enrichment in the list of DEGs, including oxidation–reduction related processes (GO:0055114, GO:0006979, GO:0016491, GO:0098869, GO:0072593, GO:0000302, GO:0034599, GO:0000303, GO:0000305, GO:0019430, GO:0071450, GO:0071451, among others), MAPK cascade (GO:0000165, GO:0004707), BCAA biosynthesis (GO:0009082, GO:0009081), nucleoplasm part (GO:0044451), PSII and PSII OEC (GO:0009523, GO:0009654), and RNA binding (GO:0003723).

Scavenging proteins act as anti-oxidants to mitigate damage by reactive oxygen species (ROS) that are both the products of abiotic stress and a signaling molecule in response to abiotic stress^46^. We identified 165 transcripts that were annotated as various ROS scavenger proteins and ROS-generating enzymes. Under the compound stress, there were 20 up-regulated transcripts compared to controls, while only two were up-regulated under HT stress, and 15 were up-regulated under LW stress. For some transcripts, expression in the compound stress was the cumulative effect of expression under HT stress and LW stress. However, the combination of the two stresses evoked a stronger response from some transcripts. Two putative alternative oxidase (AO) transcripts were up-regulated > 4 log2- fold under the compound stress that are not significantly up-regulated under HT or LW stress alone.

### DEG in root tissue

Root tissue exposed to LW stress was analyzed separately due to degradation of the RNA during processing. Root tissue exposed to CW had similar quality to leaf tissue, and thus could be compared to leaf tissue, though not to root tissue exposed to LW.

### DEG in root tissue: high temperature stress with control water (CT/CW × HT/CW)

There were 3555 transcripts that were up-regulated under HT in roots, and 4220 transcripts that were down-regulated. There were 738 GO terms over-represented in the DEGs, including regulation of biological process (GO:0050789), organonitrogen compound metabolic process (GO:1901564), regulation of nitrogen compound metabolism (GO:0051171), and oxidation–reduction process (GO:0055114). Among the list of DEG are putative peroxidases, and a number of heat shock proteins. Of the transcripts associated with response to heat (GO:0009408), most were expressed more in leaf tissue, particularly leaf tissue exposed to both LW stress and HT stress. Several hypothetical proteins, a respiratory burst oxidase (RBO) and annexin were expressed at higher levels in root tissue than in leaf tissue.

### DEG in root tissue: high temperature stress with low-water stress (CT/LW × HT/LW)

Most of the DEG were down-regulated under the compound stress; we detected 391 up-regulated transcripts, and 882 down-regulated transcripts. There were 1007 significant GO terms, including gene expression (GO:0010467), RNA processing (GO:0006396), organonitrogen compound metabolic processes (GO:1901564), response to toxic substance (GO:0009636), oxidoreductase activity (GO:0016491), antioxidant activity (GO:0016209), and cytoplasmic part (GO:0044444). Among the transcripts most up-regulated are a number of heat shock proteins, an annexin, a terpene synthase, and the nuclear transcription factor Y subunit C. There are several putative peroxidases that are significantly down-regulated under compound stress in root tissue compared to CT/LW. We also identified a putative root cap protein and a putative Casparian strip membrane protein that are significantly down-regulated under compound stress.

### Pathway analysis

**Figure 6:**
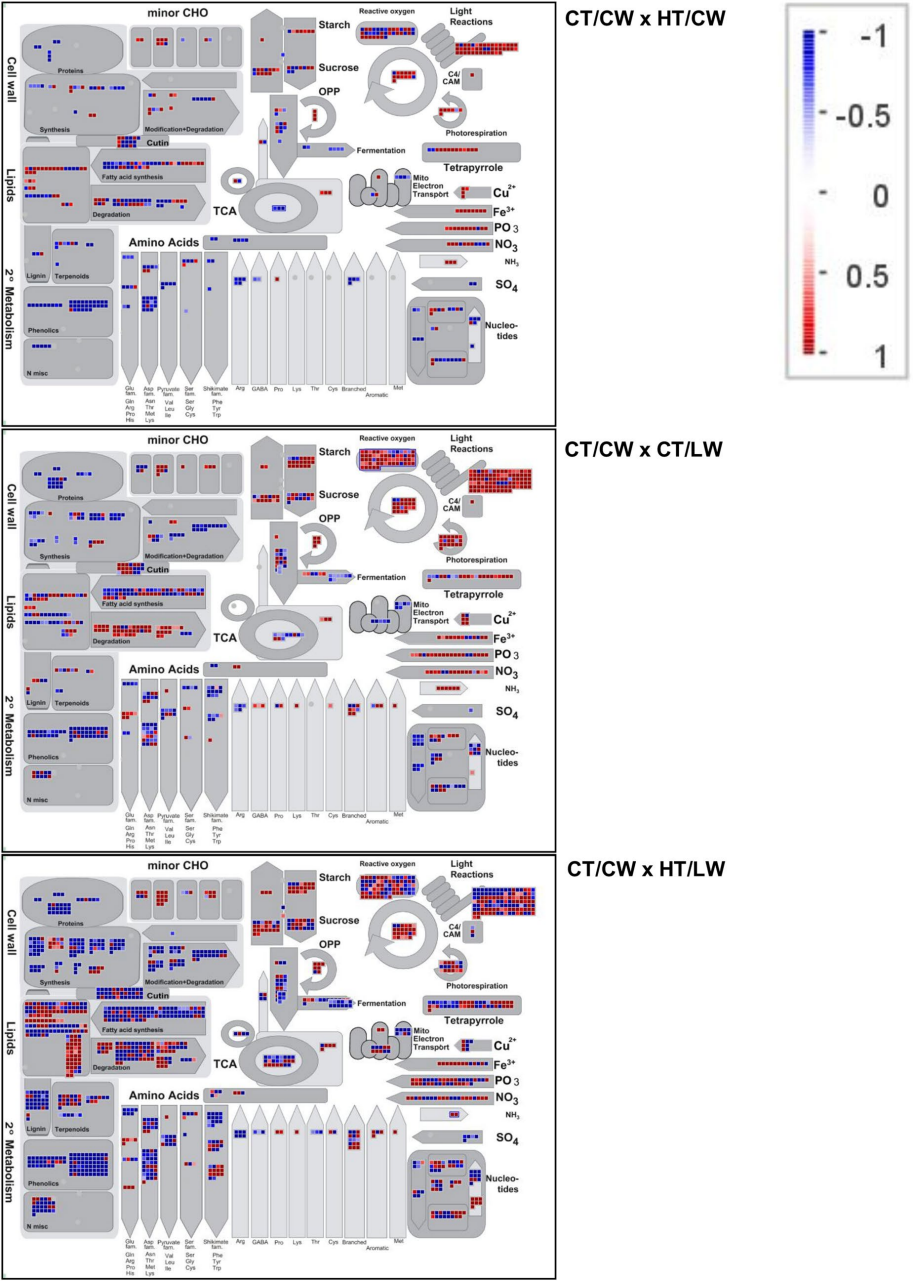
An overview map of primary metabolism pathways in leaf tissue affected by HT stress (HT/CW), LW stress (CT/LW), and the compound stress of HT and LW (HT/LW) compared to controls (CT/CW). Boxes represent a transcript annotated within each pathway, and color represents the relative degree of log2-fold change in comparison to control samples.

The metabolism overview maps show the trend toward increasing stress from HT to LW, to the compound stress (HT/LW) (Fig. 6). Transcripts putatively involved in photosynthesis and light reactions are up-regulated under HT/CW compared to controls, as are a number of transcripts involved in carbon assimilation, photorespiration, and lipid metabolism, as well as metabolism of copper, iron, phosphorus, and nitrogen. Transcripts involved in secondary metabolism pathways and BCAAs, which include transcripts in the bitter acid production pathways, are largely down-regulated under HT. This pattern of down-regulation continues with a greater number of transcripts involved in CT/LW treatments. A large number of transcripts involved in lipid degradation pathways are upregulated under LW treatments. The effect of the compound treatment is greater than cumulative in most pathways, particularly in the secondary metabolism pathways. Transcripts involved in the light reactions and copper, iron, phosphate, and nitrate metabolism are down-regulated under the compound stress (Fig. 6).

**Figure 7:**
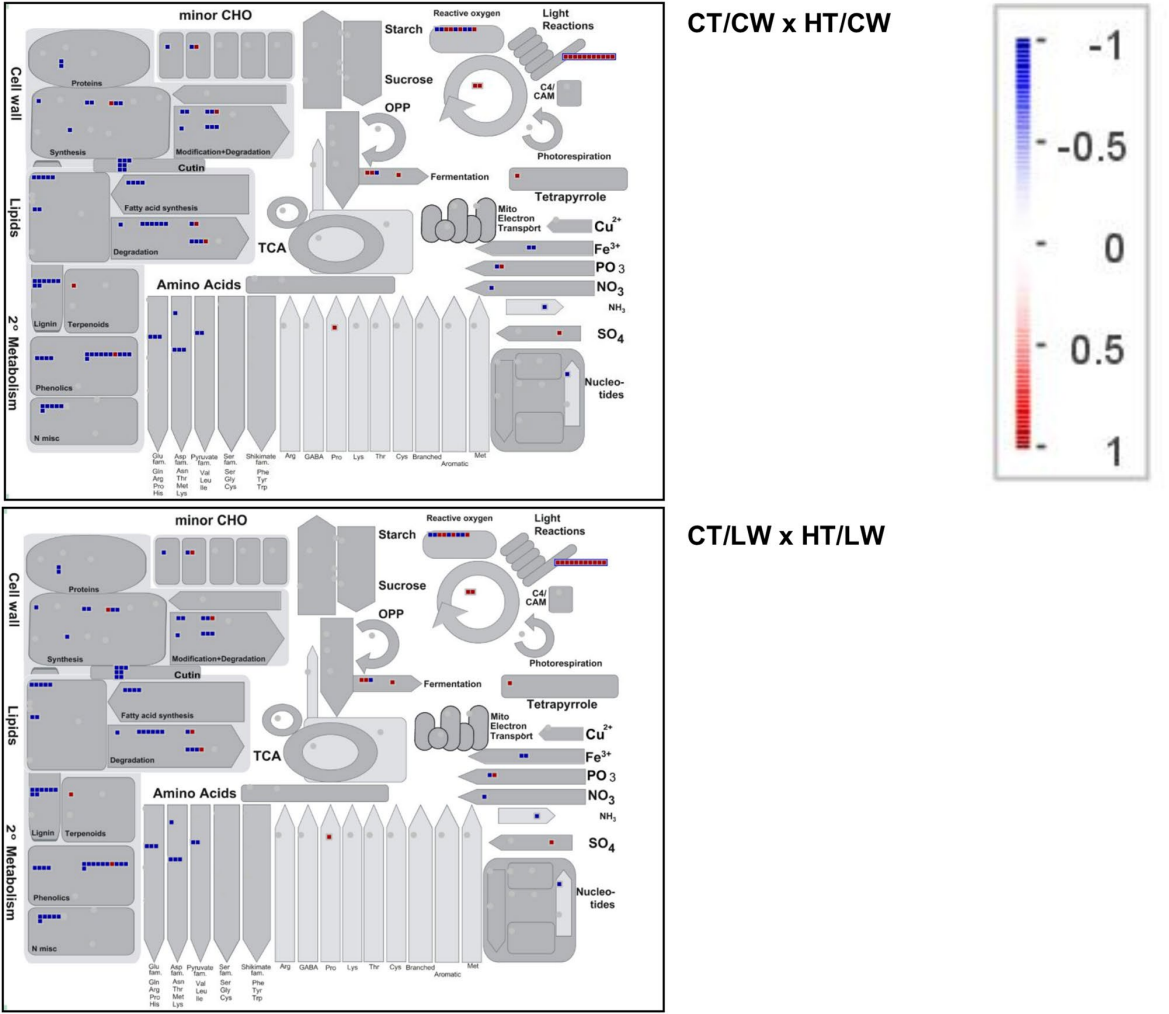
An overview map of metabolism pathways in root tissue affected by HT stress. Boxes represent a transcript annotated within each pathway, and color represents the relative degree of log-fold change in comparison to the control temperature (CT) treatment. Comparisons among CW and LW treatments were not possible due to differences in library preparation.

In root tissue, where we could only analyze samples within the same water treatment, there were not considerable differences among samples exposed to HT. The comparison of transcripts mapped to genes involved in primary metabolism show that response to HT in the roots is similar, regardless of water treatment (Fig. 7).

## Discussion

The majority of hops in the United States are grown in the Yakima Valley of the Pacific Northwest, and models predict a decrease in winter precipitation and an increase in the frequency of heat waves in Washington state in upcoming decades^7^. There is increasing interest among growers and industry partners to better understand the response of hops to HT and LW stress, and to breed varieties with improved tolerance to abiotic stress. There are a number of genomic resources now available to assist breeding for *H. lupulus*, including the genome sequence^40,47,48^, a proteome^49^, and transcriptomes^13,14^. This study adds the transcriptome sequence under a combination of HT stress and LW stress, as well as a transcriptome from hop root tissue. Our goal was to describe the response of hops to HT stress, LW stress, and a combination of these stresses in order to identify candidate genes for screening in established cultivars and new breeding lines.

The agronomically-important bitter acids are secondary metabolites that are synthesized in lupulin glands. Lupulin glands are found in cone, leaf, and stem tissues, however cone lupulin glands are the most relevant. One of the major difficulties faced in this project was the generation of sufficient hop cone tissue for RNA-seq from potted plants maintained in growth chambers, particularly under the compound HT/LW stress, which caused very severe declines in biomass. Hops generally require a minimum number of nodes prior to onset of flowering^50^ and plants exposed to HT/LW in this study did not reach this stage. Therefore, we used leaf and stem tissue and their associated lupulin glands as proxies for cone lupulin glands. The bitter acids are found in leaf tissue in small amounts^12,13^. Clark et al.^14^ and Mishra et al.^13^ found many genes for the bitter acid pathway expressed in leaf tissue, though some, including *VPS* and *BCAT2*, have higher expression in lupulin glands extracted from cones. Some genes appear to be expressed only in cone lupulin glands, such as the prenyltransferase genes *HlPT1L* and *HlPT2* and *BCAT1*^14,22^*,* and consequently these were not detected in our study. Although extrapolation of expression patterns in cone lupulin glands through leaf lupulin glands is not ideal and may not represent physiological conditions in the field, there is correlation of gene expression in these two types of lupulin glands^14^, such that we believe leaf lupulin gland expression can be used to estimate and develop hypotheses about cone lupulin gland expression.

Of the transcripts involved in bitter acid production that are expressed in leaf lupulin glands, VPS was extremely sensitive to stress treatments. Expression of the putative VPS transcript 001329F.g74 was much higher than expression of 00239F.g29, but both transcripts declined significantly in all treatments compared to control treatments, more so than the other genes involved in bitter acid metabolism. Given that there are no transcripts of *HlPT1L* and *HlPT2* in our database, we cannot link previously described declines in alpha acid content^8–10^ due to HT and LW stress exclusively to VPS. However, stress induced lability in this gene and/or its regulatory elements^51–53^ likely contributes significantly to declines in bitter acid content during periods of heat and low-water stress.

There was significant down-regulation under HT and the compound stress for many transcripts related to terpene synthesis, particularly a number of putative humulene synthases. The genes related to thiol production in hop are unknown, however glutathione S-transferase (GST) and gamma-glutamyl transferase (GGT) are known to be involved in production of grape varietal thiols^37,38^. Glutathione S-transferases are a large superfamily in plants, and many transcripts in *Arabidopsis* experience no changes in expression levels in response to phytohormones, oxidative stress, herbicide application, or pathogen inoculation^54^. In grape, UV-C irradiation and pathogen inoculation increased content of 3MH-precursors as well as expression of several GST genes in leaf tissue^37,38^. Some studies have found LW stress increases content of 3MH in grape^37^, while others have found no effects of water status on 3MH content^55^. We identified 17 transcripts as putative GSTs and five transcripts as putative GGTs, and most experienced no significant or substantial change in expression levels among treatments imposed here. At this time, however, it is not clear which GST transcripts are involved in thiol production in hop cones. Sixteen of the putative GST transcripts have significant homology to grape GST1 (NM_001281248.1), and only one is a best match to grape GST4 (NM_001280940.1); GST1 is not apparently involved in production of 3MH, though GST4 likely is involved^37^. It is also not clear how expression of transcripts putatively involved in varietal thiol and terpene biosynthesis is correlated in leaf and cone tissue. Further work is necessary to evaluate correlation in expression among leaf and cone tissue for these genes, and to identify the genes involved in varietal thiol biosynthesis in hop.

Hops appear to reach maximum carbon assimilation (A) rates at slightly higher temperatures than most plants^56^, but previous studies have not disentangled the effects of HT and LW stress. The HT stress in this study (39˚C) was at the upper level of peak A in hops, and not unusual or particularly excessive in many hop growing regions. Plants exposed to HT/CW had similar DW and physiological traits as plants in CT/CW treatments. Indeed, these plants had higher A than plants in CT/CW treatments, but there was a significant response in the transcriptome. Of the 1869 DE transcripts from comparisons of CT/CW × HT/CW plants, many can be putatively linked to alpha acid and volatile secondary metabolite production. High temperatures have been clearly linked to reductions in alpha acid production in the past^8,10^, and this study found VPS was down-regulated under heat stress. GO terms related to naringenin-chalcone synthase activity (involved in xanthohumol biosynthesis) and terpene synthase activity (involved in volatile secondary metabolite production) were enriched in the list of DE transcripts, and usually significantly down-regulated under HT stress, suggesting that xanthohumol biosynthesis and volatile secondary metabolite or “hop oil” biosynthesis are also compromised during HT stress conditions.

Photosystem II (PSII) is a primary site of damage to the photosynthetic system due to heat stress^41^. A decrease in activity of PSII and photoinhibition ensues when more damage to PSII occurs than can be repaired^57^. Damage to PSII is indicated by F_V_/F_M_ ratios. We recorded significantly decreased F_V_/F_M_ ratios under HT treatments, but the level of photoinhibition was not severe, nor sufficient to cause declines in measured A, ɸ_PSII_, or ɸ_CO2_ under HT/CW treatments. Among transcripts annotated as related to maintenance and repair of the PSII oxygen evolving complex (OEC), and non-photochemical quenching, we found a number of transcripts that were up-regulated under HT stress compared to controls, and many of which are related to stabilizing lipids. There was an increase in expression of several putative D1 protein-coding transcripts, which suggests PSII sustains some damage due to heat, but repair mechanisms were sufficiently up-regulated so that damage was not excessive enough to cause severe declines in F_V_/F_M_ and photoinhibition. Tolerance to HTs may also be due, in part, to observed up-regulation of Rubisco activase under HT stress. Eriksen et al. found relatively high *V*_*c,max*_ in cv. Cascade at 39 °C^56^, which could be achieved in part by high concentrations of Rubisco activase. Variation in Rubisco activase concentrations along latitudinal gradients in black spruce^58^ and red maple^59^ appear to impart increased temperature tolerance in these species. Inhibition of carbon assimilation during HT stress is in part attributed to denaturation of Rubisco activase^60^.

The expression levels of transcripts expressed in both leaf and root tissue tended to be higher in leaf tissue, possibly due to higher numbers of living cells in leaf tissue. However, we observed higher numbers of transcripts of a putative annexin gene in root tissue than leaf tissue. Annexins are a conserved protein family found across a diverse group of organisms^61^, and have been implicated in increasing tolerance to HT stress in rice by promoting expression of antioxidant scavengers superoxide dismutase (SOD) and catalase (CAT)^62^.

A number of studies have correlated reduced cone yield with LW stress^8,10,11^. We found significantly decreased DW, A, *g*_*sw*_, E, C_*i*_, and ɸCO_2_ under LW stress. Kolenc et al. looked at physiological traits of cv. Aurora and cv. Savinjski Golding under progressive drought, and found significant decreases in A, E, *g*_*sw*_, ETR, and F_V_/F_M_^63^. Reductions in *g*_*sw*_ and E and concomitant reductions in C_*i*_ and A are due to stomatal closure, which is achieved via ABA signaling. A critical step in the ABA signaling pathway during LW stress is activation of bZIP transcription factors. Overexpression of the bZIP transcription factor AREB1 has been associated with increased LW stress tolerance^64^, and overexpression of various bZIP transcription factors have been shown to increase LW stress tolerance in a number of crops^65–69^. We identified six transcripts with significant homology to bZIP transcription factors that are up-regulated under LW stress, and may be markers for increased tolerance to LW stress.

We also found putative nuclear transcription factor Y subunit A transcripts had greater than threefold increase in expression under stress treatment in comparison with control treatments. The protein product of this gene physically interacts with ABA-responsive bZIP transcription factor ABA-INSENSITIVES, and over-expression of this gene confers salt and osmotic hypersensitivity in *Arabidopsis*. It is a positive regulator of ABA signaling^70,71^, and higher expression of this gene may also be a potential marker for selection of abiotic stress tolerance.

ABA-independent pathways are mediated through members of the AP2/ERF family of transcription factors, DREB2A and DREB2B. DREB2 genes have increased expression levels under osmotic stress caused by LW or hypersaline conditions^72,73^, but is tightly regulated by the transcriptional repressor GRF7 (growth-regulating factor 7) under non-stress (i.e. control) growth conditions. We found significant up-regulation of several putative DREB2 transcripts, and down-regulation of a putative GRF7 under low-water stress.

The physiological response to the compound stress of HT and LW involves responses to two different stress factors that are in some ways mutually exclusive: high leaf temperature can be cooled by transpiration through open stomata, but stomata close under LW stress to preserve water. Plants exposed to HT and LW stress experience high respiration, low carbon assimilation, low stomatal conductance, and high leaf temperature, and starch breakdown is high^46,74^. Likewise in *H. lupulus*, the combined stress of HT/LW elicited a different response than either stress alone, and had a much more pronounced effect on the physiology and the transcriptome of the plants. Bine DW and A were significantly reduced in HT/LW plants, as was *g*_*sw*_, E, ETR, qP, and the indicator of photoinhibition, F_V_/F_M_.

The compound stress significantly reduced expression of transcripts in the bitter acid pathway. Twelve transcripts putatively involved in this pathway were significantly down-regulated under the compound stress, and expression of the critical gene for bitter acid production, VPS, was reduced by > 6log2-fold. Several transcripts coding for putative BCAT2 proteins were down-regulated by > 6- and > 10log2-fold under the compound stress. Two putative humulone synthases transcripts were also down-regulated by greater than > 6log2-fold.

Several ROS scavenging proteins were up-regulated under the compound stress, specifically a number of alternative oxidases (AO). Alternative oxidases are mitochondrial membrane-bound proteins that function in the electron transport chain to provide an alternative, non-energy producing, terminal oxidase for electrons, and are used as an indicator of oxidative stress^75^. These may be a symptom of stress rather than a potential marker for breeding hops that are more tolerant of abiotic stress, but may be useful as an indicator of oxidative stress in breeding lines.

## Conclusions

RNA-seq analyses are hypothesis-generating studies. With their large scale, they are intended to elucidate general trends and to indicate potential target pathways and genes for future studies. This study, though done with leaf and root tissue from *H. lupulus* rather than the agronomically more important cone tissue, suggests that genes involved in agronomically important secondary metabolite biosynthesis, particularly bitter acid biosynthesis, are affected by HT stress, LW stress, and a combination of both stress factors. The critical gene *VPS* appears more sensitive than other genes in the bitter acid pathway, however we did not recover transcripts of down-stream prenyltransferases and cannot describe their response to HT and LW stress. We also found that transcripts involved in terpene and thiol precursor biosynthesis pathways can be affected by HT and/or LW stress, though it is not clear how expression of these genes within cone lupulin glands and leaf lupulin glands correlate, or if our findings in leaf lupulin glands are relevant to extrapolate hypotheses about the effects of these stress factors on volatile secondary metabolite, or “hop oil” production. Previous studies have found that VPS expression correlates to bitter acid content^25^. Our findings relating to expression of genes in bitter acid pathways agree with previous studies that found reduced alpha acid content in hops exposed to HT stress^8–10^. Other studies that found no reductions in alpha acid content under LW stress^11^ or cultivar-specific reactions to LW and HT stress^10^ suggest cultivar differences in the temperature tolerance range of VPS and/or its regulatory mechanisms may be exploited to develop breeding lines with increased resilience to abiotic stress. Though plants grown in growth chambers under the conditions described here are very different than plants grown in the field, we anticipate that our findings will be helpful for breeding programs to identify traits and genomic regions for selection of new hop cultivars with more tolerance to abiotic stress such as HT and LW.

## Materials and methods

### Experimental treatments, phenotyping, statistics

We excavated rhizomes from three plants of the cultivar Cascade (plant “A,” “B,” “C”), which is the same cultivar from which the most complete genomic data are available^40^. Cultivars are propagated clonally, therefore all rhizomes came from genetically identical plants. Rhizome cuttings were washed thoroughly and patted dry. Cuttings were planted in pasteurized one gallon pots in sterilized potting mix (containing peat, perlite, pumice, gypsum, dolomite limestone [pH 4.0–4.5], wetting agent [moisture content 45–55%], and nutrient charge [8–15 ppm NH_4_-H, 50–100 ppm NO3-N, 16–35 ppm P, and 70–160 ppm K]) with approximately 5 cm of washed and sterilized gravel at the bottom of the pot to allow for ease of root tissue collection.

To break dormancy, all pots were placed in two growth chambers at 24 °C, the relative humidity was set to 10–20%, and plants were provided 14 h of day light at 600–875 µmol photons m^−2^ s^−1^ ambient light levels. Once dormancy broke, plants were fertilized weekly with 100 mL of a 24 N-8P-12 K fertilizer with micronutrients mixed according to the manufacturer’s instructions (MiracleGro). Starting six weeks after dormancy broke, we initiated treatments using a split-plot design, in which one growth chamber was kept at 24 °C for control temperatures, but temperatures in a second growth chamber were raised to 39 °C for HT treatments. We monitored soil moisture content using EC-5 soil moisture probes (METER Group, Pullman, WA U.S.A.), and allowed soil moisture content in nine plants per temperature treatment to drop to < 0.1 volumetric water content (VWC) m^3^/m^3^ to induce low-water (LW) stress. Soil moisture content was maintained at > 0.2 m^3^/m^3^ in water control plants. This allowed for four treatments of nine plants each: control temperature/control water/ (CT/CW), high temperature/control water (HT/CW), control temperature/low-water stress (CT/LW), and high temperature/low-water stress (HT/LW). The plants were grown under these conditions for an additional six weeks.

Using a LI-6400 Portable Photosynthesis System with a chlorophyll fluorescence sensor head (LI-COR Biosciences, Lincoln, NE U.S.A.), we measured carbon assimilation, stomatal conductance, transpiration, internal carbon concentration, and chlorophyll fluorescence traits in the second oldest and the youngest leaves two–three days before tissue collection. Flow rates on the LI-6400 were set to 300 µmol s^−1^, and a mixer was used to control CO_2_ concentrations at near atmospheric levels (400 ppm). IRGAs were matched every 30 min. For F_V_/F_M_ measurements, leaves were dark-adapted for > 30–45 min prior to measurement using leaf clips and by shutting off the growth chamber lights.

Physiological traits and growth traits were evaluated for compliance with the assumptions of parametric tests using boxplots, shapiro.test (R package stats), and levene.test (R package lawstat) in R v.3.6.1. Traits that did not meet the assumptions of normality and homoscedasticity were log- or square-root transformed to meet the assumptions, or were tested using non-parametric Kruskal–Wallis tests. We used Tukey HSD {stats} tests for post-hoc multiple comparisons in traits tested using parametric ANOVA tests, and dunn.test {dunn.test} for traits evaluated using Kruskal–Wallis tests; P-values were evaluated for significance based on a sequential Bonferroni adjusted P-value^76^.

### Tissue collection, RNA isolation, and sequencing

After six weeks of exposure to treatment, the growing tip of each plant, including apical meristems, as well as the tissue of the youngest four leaves were cut and flash frozen in liquid nitrogen. Root tissue was removed from gravel and soil and flash frozen in liquid nitrogen. Tissue from the three original, genotypically identical plants in the field (“A,” “B,” “C”) were pooled in a sample. There were thus three replicates within each treatment, and within each replicate was a pool of tissue from three plants derived from the same plant in the field. There were 12 samples of leaf and 12 samples of root tissue. Tissue was stored at − 80 °C until processing.

To isolate RNA, we ground approximately 50–100 mg tissue in liquid N, then added 600 µL extraction buffer (2.42% w/v tris base, 1.27% w/v lithium chloride, 0.37% w/v EDTA, 1.5% w/v N-lauroylsarcosine, 1.0% w/v sodium dodecyl sulfate, 1.0% w/v deoxycholic acid) with 31 µL beta-mercaptoethanol per sample and immediately vortexed. In order to ensure cell breakage, we froze the extract at – 80 °C for 10 min, then thawed at 35–40 °C for 15 min. This freeze/thaw cycle was repeated once. We then added 210 µL 8.5 M potassium acetate, and mixed by inverting. The tubes were then incubated on ice for 15 min. The tubes were centrifuged at 1950×*g* for 5 min, then again at 12,200×*g* for 5 min. The supernatant was transferred to new tubes, and 500 µL chilled PureLink Plant RNA Reagent (ThermoFisher Scientific, Waltham, MA U.S.A.) was added. From this step forward, the procedure followed the PureLink Plant RNA Reagent’s manufacturer’s instructions: we incubated the tubes at room temperature for 5 min, and then added 100 µL 5 M sodium chloride solution and mixed by inversion. We added 300 µL chloroform, mixed by inversion, and centrifuged at 12,000×*g* for 10 min at 4 °C. The tubes were then incubated on ice for three minutes to maximize chloroform separation, and the aqueous layer was transferred into a new 2 mL tube. We added 0.7 volumes of room temperature isopropyl alcohol, mixed by inversion, and incubated at room temperature for 10 min. The pellet was then washed twice with 75% cold ethanol, air dried fully, and re-suspended with 100 µL RNA-free water. Genomic DNA was removed using ThermoFisher Scientific DNase treatment according to the manufacturer’s instructions.

For 17 libraries from leaf and root tissues from control water treatments, we achieved RIN values > 8 and we used the TruSeq SR Cluster Kit v3-cBot-HS (Illumina, San Diego, CA U.S.A.) for single-end library preparation for 17 libraries. For six libraries from root tissue exposed to LW stress treatments, we used the QuantSeq 3′ mRNA-Seq Library Prep Kit FWD for Illumina (Lexogen, GmbH, Vienna, Austria) intended for low-quality samples. 100 bp sequencing took place on four lanes (three for the TruSeq SR-prepared samples, and one for the QuantSeq 3′ prepared samples) on an Illumina HiSeq 3000 at the Oregon State University Center for Genome Research and Biocomputing.

### Assembly and differential gene expression analysis of leaf tissue

Raw reads were assessed for quality using MultiQC^77^. Adaptor sequences were removed using cutadapt^78^, however poor quality bases were not removed^79^. We compared the alignment of reads against the Cascade primary coding sequences (draft form) and against the masked reference genome assembly^40^. The alignment against the draft primary coding sequences was done with Salmon^80^, with the number of aux model samples and pre aux model samples set to 100,000. For the alignment against the reference genome, we used hisat2^81–83^ with samtools^84^ and Stringtie^82,85^ to align the reads. The mapping rate was higher using the masked reference genome. Transcripts were recovered using gffread^86^, and submitted to OmicsBox for functional annotation and BLAST2GO assignments^87–89^. Annotations were cross-referenced and confirmed with Mercator4^90^.

Differential gene expression and a normalized counts of reads were calculated using DESeq2^91^. Genes listed as differentially expressed (DE) have a B-H adjusted P-value less than 0.05, and the absolute value of the log2-fold change was greater than two. We used the R package vennDiagram^92^ for Venn Diagrams. We used the R package topGO with classic Fisher exact tests to identify significantly enriched GO terms among lists of differentially expressed genes (DEGs). Heatmaps for gene expression were created using ggplot2^93^ using normalized read counts from DESeq2. Transcripts for which all gene counts were below 10 were eliminated from the heatmaps.

### Assembly and differential gene expression analysis of root tissue

Transcript assembly for root tissue exposed to control water (CW) treatments (i.e. CT/CW, HT/CW) was done as described above for leaf tissue. The libraries for root tissue for low-water (LW) treatments (i.e. CT/LW, HT/LW) were constructed using the QuantSeq 3′ mRNA-Seq Library Prep Kit FWD for Illumina (Lexogen GmbH, Vienna Austria) for degraded tissue. Adapter sequences were trimmed, while requiring a minimum read length of 20 bases using cutadapt version 1.15^78^ RNA-seq reads were then aligned to the reference assembly of Cascade^40^. We did not apply a minimum PHRED score threshold. We tested multiple programs, including STAR 2.7.1a^94^ and hisat2, to obtain the best alignment based on the mapping rate. We selected the alignment produced by STAR because the average unique mapping rate across six replicates was higher (55%) than with hisat2 (44%). The resulting bam file from STAR was sorted by coordinate using samtools^84^, and the alignments were assembled into transcripts with StringTie v1.3.3b^82^. The replicate transcript assemblies were merged with cuffmerge^95^. Transcript structure and expression levels were visualized with Integrated Genomics Viewer (IGV)^96^. Similarity to Pfam protein domains was assessed with hmmscan^97^. The differential gene expression analysis and functional annotation were performed as described above for leaf tissue.

### Annotations and pathway assembly

The transcripts were uploaded to Mercator4^90^, and the resulting annotations were downloaded to MapMan^98^. The genes involved in biosynthesis of bitter acids were identified from the literature^14–18,99^ and pulled from the predicted transcript file. The names and sequences were cross-referenced using the Mercator4 and the OmicsBox annotation, and submitted to tBLASTx against the TAIR database^100^ to confirm identity. *Humulus lupulus* homologs of grape (*Vitis vinifera*) genes involved in thiol precursor biosynthesis were identified with tBLASTx searches using NM_001281248.1, EU181421.1, NM_001280940.1, XM_002280154.3.

## Data Availability

Raw library reads will be deposited at http://hopbase.org.
